# Follistatin-Like 1 Attenuation Suppresses Intervertebral Disc Degeneration in Mice through Interacting with TNF-*α* and Smad Signaling Pathway

**DOI:** 10.1155/2021/6640751

**Published:** 2021-04-10

**Authors:** Shaoyi Wang, Jianlu Wei, Jie Shi, Qiting He, Xiaocong Zhou, Ximei Gao, Lei Cheng

**Affiliations:** ^1^Department of Orthopedic, Qilu Hospital, Cheeloo College of Medicine of Shandong University, Jinan, China; ^2^Cheeloo College of Medicine, Shandong University, Jinan, China; ^3^Shandong Qianfoshan Hospital, Cheeloo College of Medicine, Shandong University, Jinan, China; ^4^Department of International Medicine, Qilu Hospital of Shandong University, Jinan, China; ^5^Nursing Theory & Practice Innovation Research Center of Shandong University, Jinan, China

## Abstract

**Background:**

Inflammation plays an important role in intervertebral disc degeneration (IDD). The protein follistatin-like 1 (FSTL1) plays a proinflammatory role in a variety of inflammatory diseases.

**Objectives:**

The purpose of this study was to investigate whether IDD could be delayed by inhibiting FSTL-1 expression.

**Methods:**

We established a puncture-induced IDD model in wild-type and FSTL-1+/- mice and collected intervertebral discs (IVDs) from the mice. Safranin O staining was used to detect cartilage loss of IVD tissue, and HE staining was used to detect morphological changes of IVD tissue. We measured the expression of FSTL-1 and related inflammatory indicators in IVD tissues by immunohistochemical staining, real-time PCR, and Western blotting.

**Results:**

In the age-induced model of IDD, the level of FSTL-1 increased with the exacerbation of degeneration. In the puncture-induced IDD model, FSTL-1-knockdown mice showed a reduced degree of degeneration compared with that of wild-type mice. Further experiments showed that FSTL-1 knockdown also significantly reduced the level of related inflammatory factors in IVD. In vitro experiments showed that FSTL-1 knockdown significantly reduced TNF-*α*-induced inflammation. Specifically, the expression levels of the inflammatory factors COX-2, iNOS, MMP-13, and ADAMTS-5 were reduced. Knockdown of FSTL-1 attenuated inflammation by inhibiting the expression of P-Smad1/5/8, P-Erk1/2, and P-P65.

**Conclusion:**

Knockdown of FSTL-1 attenuated inflammation by inhibiting the TNF-*α* response and Smad pathway activity and ultimately delayed IDD.

## 1. Introduction

Intervertebral disc degeneration (IDD) is the most common cause of low back pain and lumbar disc herniation (LDH) and has become an important public health issue, posing a serious burden on countries, society, and families [1–3]. Inflammation and oxidative stress are thought to play important roles in disc degeneration [4, 5]. Most previous studies showed that IDD was a passive process: the local homeostasis of intervertebral disc (IVD) tissues was destroyed, the biomechanical relationship of normal lumbar vertebrae was changed, and the number of local inflammatory cells and the secretion and synthesis of related inflammatory factors were altered [4, 6–11]. The significantly high expression of inflammatory factors is an important characteristic of the inflammatory microenvironment of the degenerative nucleus pulposus. Therefore, it is critical to block the effects of inflammatory factors in IDD, inhibit the inflammatory response caused by these factors, and delay the degeneration of nucleus pulposus cells to reduce the clinical symptoms of patients.

TNF-*α* is a member of the tumor necrosis factor superfamily. As a powerful inflammatory cytokine, TNF-*α* plays an important role in the inflammatory response to degenerative diseases [12–14]. Studies have shown that the expression level of TNF-*α* is positively correlated with the degree of disc degeneration [15]. TNF-*α* levels in IVDs are associated with clinical symptoms of low back pain in patients [16]. On the one hand, TNF-*α* can exacerbate the inflammatory response by promoting the secretion of inflammatory factors such as COX-2 (cyclooxygenase-2), iNOS (inducible nitric oxide synthase), and IL-1*β* (interleukin-1*β*); on the other hand, TNF-*α* can accelerate matrix destruction by promoting the secretion of matrix-degrading enzymes such as matrix metalloproteinase- (MMP-) 13 and a disintegrin and metalloproteinase with thrombospondin motifs- (ADAMTS-) 5 [17, 18]. The NF-*κ*B and Erk signaling pathways are key pathways that facilitates the function of TNF-*α*, and their role in disc degeneration has been extensively studied [12, 19, 20]. Therefore, finding a cytokine that can specifically inhibit TNF-*α* to reduce the inflammatory response is worth exploring.

The protein follistatin-like 1 (FSTL1), also known as transforming growth factor-stimulated clone 36 (TSC-36) or follistatin-related protein (FRP), is a soluble secreted extracellular glycoprotein that plays an important role in many kinds of tissue degeneration and autoimmune diseases [21, 22]. Studies have shown that FSTL1 can alleviate the occurrence of inflammatory pulmonary fibrosis, suggesting that FSTL1 may be closely related to the occurrence of inflammation [23]. Further studies have reported that FSTL-1 regulates the secretion of inflammatory factors such as interleukin-*α*, TNF-*α*, and ADAMTS and participates in the immune inflammatory response in tissues in many systemic autoimmune diseases, including systemic lupus erythematosus, ulcerative colitis, rheumatoid arthritis, and Sjogren's syndrome [24, 25]. In rheumatoid arthritis and osteoarthritis, FSTL1 exacerbates inflammation by increasing the expression of inflammatory factors and promoting synovial proliferation by activating the NF-*κ*B signaling pathway [25]. In many previous studies, FSTL-1 can activate P-Smad1/5/8 by binding to the BMP4 receptor, causing an inflammatory response and cell damage [26, 27]. Thus, we hypothesized that FSTL-1 plays a similar role in IDD.

Herein, we specifically reduced the expression of FSTL-1 to observe the degree of disc inflammation and degeneration. Additionally, we examined the role of FSTL-1, as well as the involved pathways.

## 2. Methods

### 2.1. Mice

All animal studies were performed in accordance with institutional guidelines and approved by the Laboratory Animal Centre of Qilu Hospital of Shandong University. Male 8- to 12-week-old FSTL-1-knockdown (FSTL-1+/-) mice (Jackson Laboratories, USA) and 2-, 4-, and 9-month-old C57/BL6 wild-type (WT) mice (Qilu College of Medicine, Shandong University) were used for our experiments. All experimental animals were genotyped before use.

### 2.2. Primary Cell Isolation and Culture

We selected 2-month-old FSTL-1+/- and WT male mice to extract nucleus pulposus cells. The entire thoracolumbar spine was completely separated. The nucleus pulposus and annulus fibrosus were carefully separated under the microscope. Then, the cells were washed with sterile phosphate-buffered saline (PBS) 3 times and digested with 0.25% trypsin (Sigma, St. Louis, USA) for 30 minutes and 0.2% type II collagenase (Sigma, St. Louis, USA) for 4 hours. The nucleus pulposus cells were cultured in DMEM/F12 (HyClone, Logan, USA) supplemented with 10% foetal bovine serum (FBS; Gibco, USA), 100 U/ml penicillin, and 0.1 mg/ml streptomycin (HyClone, USA) and incubated in standard conditions (37°C, 5% CO_2_) for experiments. The culture medium was replaced every 3 days. When the nucleus pulposus cells were almost 80% confluent, the cells were subcultured at a ratio of 1 : 3. Cells up to five generations old were used in all in vitro experiments. And all experiments were performed with two repeating holes.

### 2.3. Cell Treatments

To examine the effect of FSTL-1 on IVD inflammation, mouse nucleus pulposus cells were divided into the WT group and the FSTL-1+/- group. And the two groups were stimulated with TNF-*α* (10 ng/ml) [6, 20]. The cells were harvested for real-time PCR (RT-PCR) after stimulation for 8 hours and harvested for Western blot analysis after stimulation for 48 hours [20]. Protein samples were stored at -20°, and RNA samples were stored at -80° and analyzed within 1 week.

### 2.4. Mouse Model of Puncture-Induced Disc Degeneration

Eight- to twelve-week-old FSTL-1+/- and C57/BL6 WT mice were used in this study. The 15 mice were divided into three groups (FSTL-1+/- group, WT group, and the control group). The mice were anesthetized with pentobarbital. X-ray (Siemens, Germany) radiography was used to locate the L5 and L6 discs which to be punctured. A 0.5 cm incision was made at the site of the puncture disc. After disinfection, the discs needed to be exposed were exposed posterolateral under a microscope. The disc was inserted about 0.5 mm with a 29G needle and moved out after 2 seconds [20, 28]. Puncture was performed in the FSTL-1+/- group and WT group. The control group only exposed the intervertebral disc. All the mice were housed under normal conditions. The mice were fed in a cage and rubbed with iodine every day. Biting incisions were avoided.

### 2.5. Immunohistochemistry (IHC)

At 1 week after puncture, mouse disc tissues at the site of the previous puncture (L5 and L6) were harvested and fixed with 4% paraformaldehyde for 48 hours and then decalcified with 10% ethylenediaminetetraacetic acid (EDTA) for 14 days. The IVDs were then made into 5 *μ*m paraffin sections. The sections were treated with 0.125% trypsin (ZSGB-Bio, Beijing, China) for 30 minutes at 37°C for antigen repair, 3% hydrogen peroxide for 20 minutes at room temperature to eliminate endogenous peroxidase activity, and 20% goat serum (ZSGB-Bio, Beijing, China) for 20 minutes to block the nonspecific protein binding sites. Then, the sections were incubated with goat anti-FSTL-1 (1 : 200, Abcam, USA), rabbit anti-COX-2 (1 : 200, Abcam, USA), rabbit anti-ADAMTS-5 (1 : 200, Abcam, USA), and rabbit anti-MMP-13 (1 : 200, Abcam, USA) at 4°C overnight. The next day, the sections were incubated with mouse anti-goat immunoglobulin- (IgG-) horseradish peroxidase (HRP) (1 : 200, *Jackson* ImmunoResearch, USA) or goat anti-rabbit IgG-HRP secondary antibody (1 : 200, *Jackson* ImmunoResearch, USA) at 37°C for 1 hour. The results were observed with an IX71-SIF microscope (Olympus, Tokyo, Japan). Brown particles were considered positive, and the relative area of brown particles was used for the Image-Pro Plus 6.0 software (Media Cybernetics, Inc., USA) analysis.

Safranin O/Fast Green staining was performed with a modified Safranin O/Fast Green FCF cartilage stain kit (Solarbio, Beijing, China) according to the manufacturer's instructions.

### 2.6. Immunofluorescence Staining

After treatment with 10 ng/ml TNF-*α* for 48 h, nucleus pulposus cells were fixed with 4% formaldehyde for 15 minutes, permeabilized in 0.2% Triton-X 100 for 15 minutes, and blocked in 1% BSA for 30 minutes. Then, the cells were incubated with rabbit anti-ADAMTS-5 (1 : 400, Abcam, USA) and rabbit anti-COX-2 (1 : 400, Abcam, USA) primary antibodies overnight. The next day, the cells were incubated with fluorescently labelled goat anti IgG (1 : 200, ZSGB-BIO, China) for 1 hour. The results were observed by a microscope (Olympus IX51, Japan). Green fluorescence is considered positive. The Image-Pro Plus 6.0 software (Media Cybernetics, Inc., USA) was used for quantitative analysis of pictures.

### 2.7. Total Protein Extraction and Western Blotting

The nucleus pulposus tissue harvested from mice and nucleus pulposus cells were washed with sterile PBS 3 times and placed in RIPA lysis buffer (Millipore, Billerica, MA, USA) supplemented with 5% PMSF (a protease inhibitor) on ice for 40 minutes. After centrifugation at 12,000 rpm for 15 minutes at 4°C, the supernatant was retained. A BCA protein assay kit (Biotechnology Co, Beijing, China) was used to measure the protein concentration according to the manufacturer's instructions. Equal amounts of proteins were resolved on 10% SDS-polyacrylamide gels and transferred to a polyvinylidene difluoride (PVDF) membrane (Millipore, USA). After being blocked in Tris-buffered saline with Tween-20 (TBST) with 5% milk powder, the blots were incubated with goat anti-FSTL-1 (1 : 1,000, Abcam, USA), rabbit anti-COX-2 (1 : 1,000, Abcam, USA), rabbit anti-ADAMTS-5 (1 : 1,000, Abcam, USA), rabbit anti-MMP-13 (1 : 1,000, Abcam, USA), rabbit anti-iNOS (1 : 1,000, Abcam, USA), rabbit anti-P-smad2/3(1 : 1,000, CST, USA), rabbit anti-P-smad1/5/8 (1 : 1,000, CST, USA), rabbit anti-P-Erk1/2(1 : 1,000, CST, USA), rabbit anti-P-P65(1 : 1,000, CST, USA), and rabbit anti-GAPDH-HRP (1 : 5,000, ProteinTech, USA) primary antibodies overnight at 4°C. GAPDH was used to ensure equal protein loading. After being incubated with goat anti-rabbit IgG-HRP (1 : 3000, *Jackson* ImmunoResearch, USA) or mouse anti-goat IgG-HRP secondary antibody (1 : 3000, *Jackson* ImmunoResearch, USA) for 1 hour at room temperature, the blots were visualized using a chemiluminescence system (Amersham Imager 600, GE Amersham USA), and grey value analysis was used with the ImageJ software (National Institutes of Health, USA).

### 2.8. RNA Extraction and Real-Time PCR

Mouse nucleus pulposus tissues (2-3 mg) and nucleus pulposus cells were treated with TNF-*α* and lysed with TRIzol reagent (Takara Bio, Japan). A total of 1 *μ*g of RNA was used for reverse transcription by a cDNA synthesis kit (GeneCopoeia, Inc., USA). RT-PCR was performed in 10 *μ*l of SYBR Green PCR matrix mix (Toyobo, Japan) on a thermal cycler (Bio-Rad, Hercules, USA). Cycle parameters were as follows: 95°C for 1 min, 40 cycles (95°C for 15 s, 60°C for 15 s, and 72°C for 45 s), and 72°C for 5 min. The RT-PCR results were calculated using the 2^-*ΔΔ*Ct^ method. The primers (FSTL-1, COX-2, MMP-13, ADAMTS-5, *β*-actin, and iNOS) were designed based on published sequences of these genes and listed in Table 1 [29, 30].

### 2.9. Statistical Analyses

GraphPad Prism 7 (GraphPad Software Inc., San Diego, CA, USA) and Statistical Package for Social Sciences version 25.0 were used for statistical analyses, including one-way analysis of variance (ANOVA) and *t*-tests. The data are expressed as the mean value ± standard deviation (SD), and *P* < 0.05 was considered significant.

## 3. Results

### 3.1. FSTL-1 Was Expressed in the Nucleus Pulposus of Mice and Was Increased with Age

**Figure 1:**
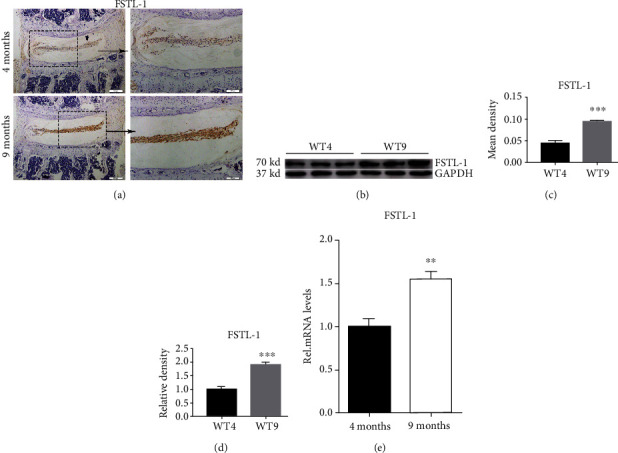
FSTL-1 was expressed in the nucleus pulposus of mice and was increased with age. (a) The expression of FSTL-1 in mouse disc tissues was determined by immunohistochemical staining. (b) Quantitative analysis of the FSTL-1-positive area in disc tissues of 4-month-old and 9-month-old mice by immunohistochemical staining. (c) Western blot analysis of the expression of FSTL-1 in mouse disc tissues. The transcript level of PGRN was measured by RT-PCR. (d) Western blot grey value analysis of FSTL-1 in the disc tissues of 4-month-old and 9-month-old mice. (e) The RNA level of FSTL-1 in 4-month-old and 9-month-old mice was assayed by RT-PCR. Magnification ×200, ×400. Scale bar = 200 *μ*m, 100 *μ*m. The values are the mean ± SD of at least 3 independent experiments; ∗∗*P* < 0.01, ∗∗∗*P* < 0.001.

To verify the role of FSTL-1 in mouse IVDs, we measured the expression of FSTL-1 in the IVDs of mice during ageing. The IHC results demonstrated that the level of FSTL-1 in IVDs was elevated in the 9-month-old group compared with the 4-month-old group (Figure 1(a)). The positive expression area and intensity of FSTL-1 in the 9-month-old group were significantly higher than those in the 4-month-old group (Figure 1(b)). We extracted the IVD tissue from mice in the two groups and performed Western blot analysis. As shown in Figures 1(c) and 1(d), the Western blot results showed that the protein expression of FSTL-1 in the 9-month-old group was higher than that in the 4-month-old group. The RT-PCR results also indicated that the transcription level of FSTL-1 in the 9-month group was higher than that in the 4-month group. These results indicated that the expression of FSTL-1 increased with age in the mouse IVD. FSTL-1 is involved in IDD in mice.

### 3.2. FSTL-1-Knockdown Mice Exhibited Reduced Cartilage Degeneration and Disc Degeneration

**Figure 2:**
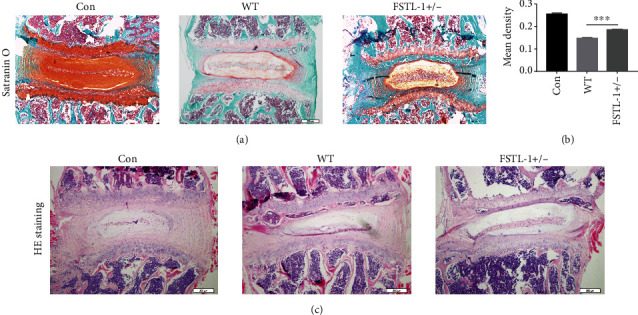
FSTL-1-knockdown mice expressed a lower level of cartilage degeneration and suppressed disc degeneration. (a) Safranin O staining of IVD tissues in the different groups. CON: normal discs. WT: degenerative discs of WT mice. FSTL-1+/-: degenerative discs of FSTL-1-knockdown mice. Magnification ×200. Scale bar = 200 *μ*m. (b) Quantitative analysis of the cartilage area based on Safranin O staining. (c) Representative image of HE staining of IVDs in the different groups. Magnification ×200. Scale bar = 200 *μ*m. ∗∗∗*P* < 0.001.

To determine the role of endogenous FSTL-1 in IDD, we used 8- to 12-week-old FSTL-1+/- and C57/BL6 WT mice to establish a model of IDD by acupuncture. After 7 days, we harvested the IVD tissue and prepared paraffin sections. The Safranin O staining results showed that IVD inflammation caused cartilage loss, but knockdown of FSTL-1 alleviated the loss of cartilage (Figures 2(a) and 2(b)). As shown in Figure 2(c), HE staining showed that inflammation accelerated the loss of disc height and caused the annulus to be disorganized in the IVD, and knockdown of FSTL-1 alleviated this structural degeneration. These results suggest that a reduction in FSTL-1 may alleviate disc degeneration.

### 3.3. FSTL-1-Knockdown Mice Had Reduced Levels of Inflammatory Cytokines in the IVDs

**Figure 3:**
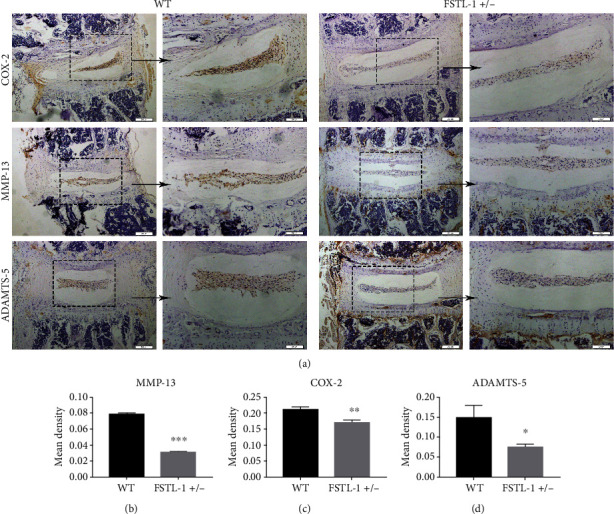
FSTL-1-knockdown mice had reduced levels of inflammatory cytokines in the IVDs. (a) Immunohistochemical staining of COX-2, MMP-13, and ADAMTS-5 in the degenerative discs of WT and FSTL-1+/- mice. Magnification ×200, ×400. Scale bar = 200 *μ*m, 100 *μ*m. (b–d) Quantitative analysis of the positive areas of COX-2, MMP-13, and ADAMTS-5 in WT and FSTL-1+/- mice by immunohistochemical staining. The values are the mean ± SD of at least 3 independent experiments; ∗*P* < 0.05, ∗∗*P* < 0.01, ∗∗∗*P* < 0.001.

**Figure 4:**
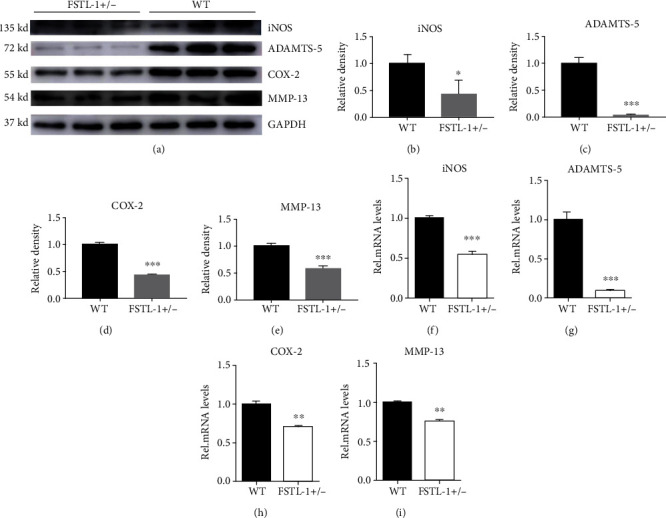
FSTL-1-knockdown mice had reduced levels of inflammatory cytokines in the IVDs. (a) The protein levels of iNOS, COX-2, MMP-13, and ADAMTS-5 in the disc tissues after puncture in WT and FSTL-1+/- mice. (b–e) Relative density of iNOS, COX-2, MMP-13, and ADAMTS-5 by Western blotting. (f–i) mRNA levels of iNOS, COX-2, MMP-13, and ADAMTS-5 in disc tissues after puncture in WT and FSTL-1+/- mice. The values are the mean ± SD of at least 3 independent experiments; ∗*P* < 0.05, ∗∗*P* < 0.01, ∗∗∗*P* < 0.001.

Inflammation is thought to play an important role in IDD. FSTL-1 plays a proinflammatory role in a variety of inflammatory diseases. Therefore, we first hypothesized that FSTL-1 knockdown probably reduced inflammatory cytokines, alleviating IDD. Therefore, we established a mouse model of puncture-induced disc degeneration and analysed IVD tissues from FSTL-1+/- and C57/BL6 WT mice after 7 days. Immunohistochemical staining showed that COX2, MMP-13, and ADAMTS-5 were expressed in the IVD during IDD, while the expression of these inflammatory factors was decreased after FSTL-1 was knocked down (Figures 3(a) and 3(b)). We further extracted the total proteins in the IVD tissue and performed Western blotting. As shown in Figures 4(a)–4(e), compared with that of the WT group, the expression of inflammatory cytokines (iNOS, COX-2, MMP-13, and ADAMTS-5) in the FSTL-1+/- group was decreased, and the difference was statistically significant. The RT-PCR results also showed that knockdown of FSTL-1 reduced the transcription level of inflammatory factors (iNOS, COX-2, MMP-13, and ADAMTS-5) in the IVD. These results suggest that knockdown of FSTL-1 can reduce the inflammatory response and delay disc degeneration.

### 3.4. Knockdown of FSTL-1 Reduced TNF-*α*-Induced Inflammatory Cytokines In Vitro

**Figure 5:**
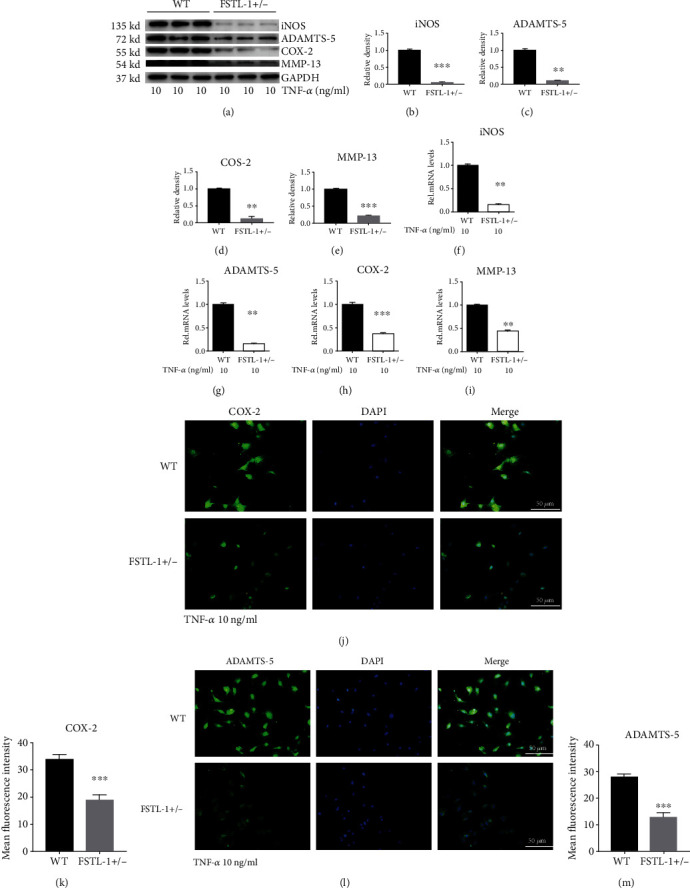
Knockdown of FSTL-1 reduced TNF-*α*-induced inflammatory cytokines in vitro. (a–e) Western blot and grey value analysis of iNOS, COX-2, MMP-13, and ADAMTS-5 in nucleus pulposus cells from WT and FSTL-1+/- mice after stimulation with 10 ng/ml TNF-*α*. (f–i) The mRNA levels of iNOS, COX-2, MMP-13, and ADAMTS-5 in nucleus pulposus cells from WT and FSTL-1+/- mice after stimulation with 10 ng/ml TNF-*α* were determined by RT-PCR. (j, l) The expression of COX-2 and ADAMTS-5 in nucleus pulposus cells after stimulation with 10 ng/ml TNF-*α* was detected by immunofluorescence analysis. Scale bar = 50 *μ*m. (k, m) Analysis of the mean fluorescence intensity of COX-2 and ADAMTS-5 according to the immunofluorescence results. The values are the mean ± SD of at least 3 independent experiments; ∗∗*P* < 0.01, ∗∗∗*P* < 0.001.

To further examine the role of FSTL-1, we extracted nucleus pulposus cells from FSTL-1+/- and C57/BL6 WT mice for in vitro experiments. Total protein was extracted after 48 hours of stimulation with 10 ng/ml TNF-*α*, and Western blotting was performed to measure the expression of COX-2, MMP-13, ADAMTS-5, and iNOS. As shown in Figures 5(a)–5(e), knockdown of FSTL-1 reduced TNF-*α*-induced inflammatory cytokines (COX-2, MMP-13, ADAMTS-5 and iNOS). Then, we extracted the total RNA and performed RT-PCR to verify the transcription levels of these inflammatory cytokines after 6 hours of stimulation with TNF-*α*. As shown in Figures 5(f)–5(i), compared with those of the WT group, the transcription levels of COX-2, MMP-13, ADAMTS-5, and iNOS were significantly decreased in the FSTL-1+/- group. After 48 hours of TNF-*α* stimulation, immunofluorescence staining of COX-2 and ADAMTS-5 was performed. As shown in Figures 5(j)–5(m), the expression of COX-2 and ADAMTS-5 was markedly downregulated in the FSTL-1+/- group. In summary, knockdown of FSTL-1 can reduce TNF-*α*-induced downstream inflammatory molecules, thereby reducing the degree of the inflammatory response.

### 3.5. Knockdown of FSTL-1 Reduced the TNF-*α*-Mediated Inflammatory Response by Inhibiting the Expression of P-Smad1/5/8, P-P65, and P-Erk1/2

**Figure 6:**
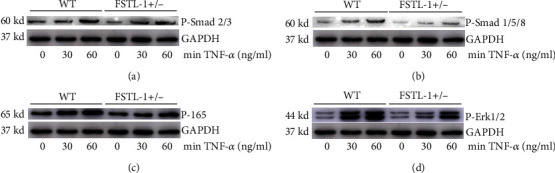
Knockdown of FSTL-1 can reduce the TNF-*α*-mediated inflammatory response by inhibiting the expression of P-Smad1/5/8, P-P65, and P-Erk1/2. (a–d) Western blot analysis of P-Smad2/3, P-Smad1/5/8, P-P65, and P-Erk1/2 in nucleus pulposus cells at different time points after stimulation with 10 ng/ml TNF-*α* in WT and FSTL-1+/- mice.

To further investigate the mechanism by which FSTL-1 knockdown inhibited the inflammatory response, TNF-*α* was used to generate an inflammatory environment in vitro, and the phosphorylation levels of corresponding pathway proteins were measured. Our results showed that the expression levels of P-Smad1/5/8, P-P65, and P-Erk1/2 could be reduced by knockdown of FSTL-1 (Figures 6(b)–6(d)). There was no significant change in P-Smad2/3 (Figure 6(a)).

## 4. Discussion

FSTL1, also known as FRP, is a secreted glycoprotein that is involved in various pathological and physiological processes, such as immune regulation, growth factors, cell proliferation, and differentiation, as well as the development of the central nervous system, orthopedic system, and respiratory system [22, 23, 31–40]. In the context of the inflammatory response, FSTL-1 has been reported to play both anti-inflammatory and proinflammatory roles [26]. In an ischemia/reperfusion-induced myocardial injury model, systemic or intracoronary injection of FSTL1 reduced the expression of inflammatory cytokines such as TNF-*α* and IL-6, thereby improving cardiac hypertrophy and dysfunction [27]. In a mouse arthritis model, FSTL-1 significantly reduced the expression of IL-6 and MMP-13, thereby reducing joint destruction and synovial inflammation [41, 42].

In contrast to the few reported anti-inflammatory effects of FSTL-1, a large number of studies have shown that FSTL-1 promotes inflammation by stimulating the release of inflammatory factors [43, 44]. Cluterr et al. showed that the upregulation of FSTL-1 resulted in significant paw swelling and upregulation of the IFN receptor, which could be neutralized by downregulating FSTL-1 [45]. In addition, Miyamae et al. transfected FSTL-1 into fibroblasts and macrophages and showed that FSTL-1 promoted the production of inflammatory cytokines, including IL-1*β*, TNF-*α*, and IL-6 [46]. Another study showed that when synoviocytes from the synovial tissues of patients with osteoarthritis were exposed to recombinant FSTL-1 protein, the levels of some inflammatory factors, such as TNF-*α*, IL-6, and IL-1*β*, were significantly increased, and the activation of the NF-*κ*B signaling pathway was also significantly upregulated [25]. FSTL-1 has also been reported to promote the transcription of NLRP3 and procaspase-1 and promote the secretion of IL-1*β* by macrophages [47]. It has been shown that oxidized low-density lipoprotein (oxLDL) increased the production and secretion of the inflammatory cytokines TLR4, IL-6, IL-8, and ICAM-1 in a model of endothelial cell injury induced by oxLDL, while the levels of these molecules completely decreased when FSTL-1 was depleted [48]. In conclusion, FSTL-1 can upregulate the expression of proinflammatory factors and promote the inflammatory response.

In summary, FSTL-1 plays a role in promoting the inflammatory response in a variety of inflammatory diseases. The upregulation of FSTL-1 at the gene or protein level can promote the release of inflammatory factors such as IL-1*β*, TNF-*α*, COX-2, MMP-13, and IL-6. Inflammation is considered an important cause of disc degeneration. Inflammatory factors can cause inflammatory responses and accelerate disc destruction by inducing nucleus pulposus cell apoptosis and matrix degradation [20, 30]. Therefore, we hypothesized that FSTL-1 plays an important role in disc inflammation. We observed increased levels of FSTL-1 in degenerated IVDs in humans and rats. In vitro, recombinant FSTL-1 increased inflammatory factor levels through the Erk1/2, JNK, and NF-*κ*B pathways [16]. Although FSTL-1 can promote inflammation, our original aim was to suppress inflammation and delay disc degeneration. To further investigate whether FSTL-1 can be a target for the treatment of IDD, we will suppress FSTL-1 and observe the degree of disc degeneration.

Previous studies have shown that ageing is an important factor in disc degeneration [30, 49]. In this study, we used longitudinal analysis to evaluate the changes in FSTL-1 in the IVD. We extracted the disc tissues of sex-matched mice of different ages and measured the expression of FSTL-1. The level of FSTL-1 in the disc tissue of 9-month-old mice was significantly higher than that of 4-month-old mice. This finding also indicates an increased level of FSTL-1 in the degenerated discs.

FSTL-1 gene-knockout (KO) mouse pups die of respiratory failure soon after birth [50]. Therefore, in this study, we used FSTL-1+/- mice. We constructed a model of puncture-induced disc degeneration, which resulted in inflammatory degeneration of IVDs. We observed that when we inhibited the expression of FSTL-1, the morphology of the mouse disc was repaired, and cartilage degeneration was alleviated. This finding suggests that FSTL-1 knockdown can protect chondrocytes and cartilage matrix. Cartilage oligomeric matrix protein (COMP) and type-2 collagen degradation of matrix proteins is an important change in IDD [17, 51–55]. MMP-13 and ADAMTS-5 have been reported to be involved in the degradation of disc matrix, exacerbating disc degeneration [20, 56, 57]. In our study, after the expression of FSTL-1 was knocked down, the expression levels of both MMP-13 and ADAMTS-5 were decreased. On the other hand, inflammatory factors such as COX-2, iNOS, and TNF-*α* can stimulate the inflammatory response, thus exacerbating the local inflammatory microenvironment, causing metabolic dysfunction of local cells, and ultimately exacerbating the degeneration of IVDs. We observed that FSTL-1 knockdown could effectively reduce the expression of these inflammatory factors. In summary, knockdown of FSTL-1 can delay disc degeneration by inhibiting both the degradation of the disc matrix and the expression of inflammatory factors.

TNF-*α*, a potent inflammatory cytokine, is highly associated with the development and degeneration of IVDs [15]. On the one hand, TNF-*α* can exacerbate the structural destruction of IVD tissue and increase the inflammatory bone absorption of adjacent vertebral bodies by inducing the expression of inflammatory factors [58]. On the other hand, TNF-*α* can upregulate IVD matrix-degrading enzymes such as ADAMTS-5 and MMP-13 through the NF-*κ*B signaling pathway, affecting the metabolic homeostasis of IVD cells and resulting in protein denaturation and necrosis [17, 56]. Therefore, how to effectively inhibit the effect of TNF-*α* in IDD is still a hot research area. In vitro, TNF-*α* was used to stimulate nucleus pulposus cells from two different sources, WT mice and FSTL-1+/- mice. Our results showed that FSTL-1 knockdown reduced the protein and transcript levels of major inflammatory factors, such as COX-2, iNOS, MMP-13, and ADAMTS-5, induced by TNF-*α* stimulation. In summary, knockdown of FSTL-1 can suppresses the TNF-*α*-mediated inflammatory response, reduce the release of inflammatory factors, and reduce the production of matrix metalloproteinases, thereby delaying the degeneration of IVDs.

Oxidative stress is also an important cause in disc degeneration [59]. In the process of oxidative stress, the mitochondria will produce excessive ROS, which can directly damage nucleus pulposus cells, interfere with the synthesis of cell matrix, and increase the expression of MMP [60]. TNF-*α* can promotes oxidative stress in a variety of disease processes [61, 62]. In intervertebral discs, TNF-*α* can induce oxidative stress in human NP cells, specifically by promoting mitochondrial swelling, lowering membrane potential, and increasing ROS levels [6]. Therefore, knockdown of FSTL-1 may not only reduce the inflammatory effect of TNF-*α* but also inhibit the effect of TNF-*α* on oxidative stress.

**Figure 7:**
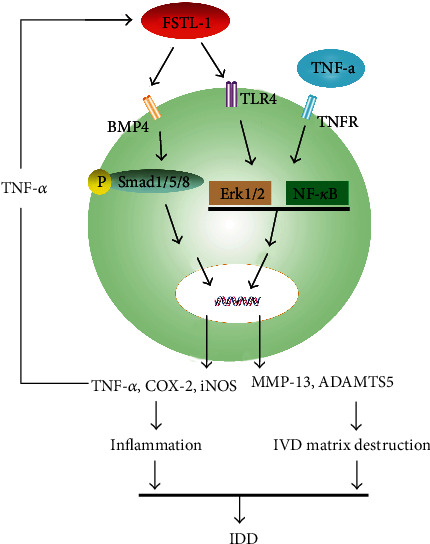
Schematic model of the proposed signaling pathways of the effect of FSTL-1 in IDD.

In osteoarthritis and disc degeneration, TNF-*α* can activate the NF-*κ*B and Erk pathways by binding specifically to TNF receptors, resulting in inflammatory changes and cartilage degeneration [30, 63]. In previous studies, FSTL-1 was reported to promote inflammatory responses through the NF-*κ*B and Erk pathways by specifically binding to TLR4 [16, 22, 25, 48]. After knockdown of FSTL-1, we found that the common pathway of FSTL-1 and TNF-*α* was also inhibited. We hypothesized that the NF-*κ*B and Erk pathways were also inhibited when FSTL-1 was knocked down, weakening the inflammatory effect of TNF-*α*. This may explain why knockdown of FSTL-1 attenuated the TNF-*α*-mediated inflammatory response. In addition, FSTL-1 can activate P-Smad1/5/8 by binding to the BMP4 receptor, causing an inflammatory response and cell damage [26, 27, 64]. Therefore, in our study, we measured the expression levels of two key P-Smad proteins. Consistent with previous reports, the expression level of P-Smad1/5/8 decreased after FSTL-1 was knocked down, while the expression level of P-Smad2/3 did not change significantly. This finding indicated that P-Smad1/5/8 was involved in the inflammatory response mediated by FSTL-1. In summary, knockdown of FSTL-1 suppressed the effect of TNF-*α* by inhibiting the activity of the NF-*κ*B and Erk pathways; on the other hand, knockdown of FSTL-1 attenuated the proinflammatory effect by inhibiting the activity of P-Smad1/5/8(Figure 7).

## 5. Conclusion

Knockdown of FSTL-1 can reduce inflammation in the IVD by reducing TNF-*α*-mediated inflammation and Smad signaling pathway, protecting cartilage, and thus delaying degeneration of the IVD in mice. FSTL-1 could be a potential target for the treatment of IDD.

## Figures and Tables

**Table 1:** Real-time PCR primers.

Target	Forward primers, 5′-3′	Reverse primers, 5′-3′
FSTL-1	TTATGATGGGCACrGCAAAGAA	ACTGCCTTTAGAGAACCAGCC
ADAMTS-5	GCATTGACGCATCCAAACCC	CGTGGTAGGTCCAGCAAACAGTTAC
iNOS	ACAGGAGGGGTTAAAGCTGC	TTGTCTCCAAGGGACCAGG
*β*-Actin	CCTCATGAAGATCCTGACCG	ACCGCTCATTGCCGATAGTG
MMP-13	ACTTTGTTGCCAATTCCAGG	TTTGAGAACACGGGGAAGAC
COX2	TCCCTTGGGTGTCAAAGGTAAA	TGGCCCTCGCTTATGATCTG

## Data Availability

The datasets used and/or analyzed of this study are available from authors on reasonable request.

## Abbreviations

FSTL-1: Follistatin-like 1
IDD: Intervertebral disc degeneration
TNF-*α*: Tumor necrosis factor *α*
IVD: Intervertebral disc
MMP-13: Matrix metallopeptidase 13
ADAMTS-5: Disintegrin and metalloproteinase with thrombospondin motifs-5
iNOS: Inducible nitric oxide synthase
COX-2: Cyclooxygenase-2
IL-1*β*: Interleukin-1*β*.
